# Joint Associations of Cumulative C-Reactive Protein–Triglyceride–Glucose Index and Depression with Cardiovascular Outcomes in Middle-Aged and Older Adults: A National Prospective Cohort Study

**DOI:** 10.5334/gh.1565

**Published:** 2026-06-17

**Authors:** Qinxian Tu, Zhuoxuan He, Yizhuo Duan, Xiongjing Jiang, Hui Dong, Yubao Zou

**Affiliations:** 1 Department of Cardiology, National Center for Cardiovascular Diseases Fuwai Hospital, Peking Union Medical College, Beijing 100037, China; 2 Department of Psychiatry, National Clinical Research Center for Mental Disorders, and National Center for Mental Disorders, The Second Xiangya Hospital of Central South University, Changsha 410011, China

**Keywords:** cumulative C-reactive protein–triglyceride glucose index, depression, cardiovascular disease, cohort study

## Abstract

**Background:**

The C-reactive protein–triglyceride–glucose index (CTI) and depression are each associated with elevated cardiovascular disease (CVD) risk. However, evidence on long-term cumulative CTI exposure and its joint effect with depression remains limited.

**Methods:**

This prospective cohort study included participants from the 2015 baseline of the China Health and Retirement Longitudinal Study, with follow-up in 2018 and 2020. Cox proportional hazards models were used to examine associations between a combined cumulative CTI–depression indicator and incident CVD, as well as their interaction. Restricted cubic splines were used to assess dose–response relationships across depression status. The predictive performance of the composite indicator was compared with individual components using integrated discrimination improvement and net reclassification improvement. Subgroup and sensitivity analyses were conducted.

**Results:**

Participants were categorized according to cumulative CTI level and depression status. Compared with individuals with low cumulative CTI and no depression, all other groups exhibited significantly higher risks of CVD, demonstrating a clear graded association. These associations remained robust after multivariable adjustment. In the primary fully adjusted model, participants with high cumulative CTI and depression had the highest CVD risk (HR = 1.83, 95% CI 1.53–2.20, *p* < 0.01). Categorical analyses suggested possible effect modification by depression status. In addition, the combined cumulative CTI–depression indicator demonstrated improved predictive performance compared with either component alone. The primary associations remained broadly consistent across subgroup and sensitivity analyses, although evidence for possible effect modification varied across alternative analytical approaches.

**Conclusions:**

The combined cumulative CTI-depression indicator was strongly and consistently associated with increased CVD risk, exhibiting graded associations with possible effect modification by depression. This joint measure captures cumulative metabolic–inflammatory burden and psychological distress and may provide complementary information for cardiovascular risk stratification beyond the individual components.

## Graphical Abstract

**Figure:**
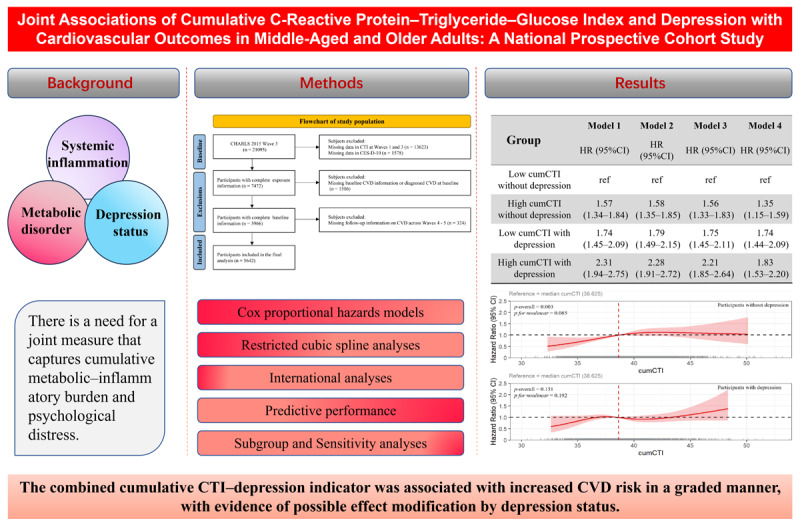


## 1. Introduction

Cardiovascular disease (CVD) refers to a broad group of disorders that affect the heart and blood vessels. It primarily encompasses coronary heart disease, cerebrovascular disease, and peripheral arterial disease, among others (1). According to the Global Burden of Disease Study, CVD continues to be the leading cause of mortality globally. Responsible for approximately 20.5 million deaths each year, it represents almost one-third of worldwide deaths (2). Beyond its impact on life expectancy, CVD may also directly or indirectly contribute to cognitive impairment or disability, leading to a substantial decline in quality of life (3,4). In the context of the growing global burden of CVD, identifying novel and quantifiable predictive markers to facilitate early risk detection and management is of substantial public health importance.

Depression is one of the most common mental disorders, affecting an estimated 280 million people worldwide and contributing substantially to the global burden of disease (5). As it is a common comorbidity of CVD, it may contribute to its development through multiple biological pathways, including inflammatory activation (6), dysregulation of the hypothalamic–pituitary–adrenal (HPA) axis (7), and autonomic nervous dysfunction (8). Growing evidence has demonstrated a significant association between depression and the onset of CVD (9,10,11). For example, Harshfield et al. analyzed multiple cohort studies and reported that for each one-standard-deviation increase in depressive symptom scores, the risk of developing CVD rose by approximately 1.06 to 1.10 times (9). A prospective cohort study reported that patients with coronary heart disease who exhibit depressive symptoms have a two- to three-fold higher risk of future CVD events compared with those without depressive symptoms (12). Meta-analyses have shown that depression is associated with an increased risk of myocardial infarction and stroke (13,14).

The triglyceride–glucose (TyG) index, proposed in 2008 as a simple biomarker for assessing insulin resistance (15), has been increasingly applied in CVD research (16,17). The C-reactive protein (CRP), a widely used nonspecific inflammatory marker, is regarded as a sensitive indicator of systemic inflammation (18). Elevated CRP levels have also been shown to be closely associated with increased CVD risk (19). The C-reactive protein–triglyceride–glucose index (CTI), proposed by Ruan et al. in 2022, is a novel composite biomarker originally developed to predict clinical outcomes in cancer patients (20), integrating both metabolic and inflammatory components. In recent years, it has garnered increasing attention for its potential clinical applicability. Prospective cohort studies have shown that CTI is significantly positively associated with CVD mortality, overall CVD risk, and all-cause mortality (21,22). Additionally, evidence from a large cohort study has reported a significant linear relationship between CTI and stroke risk, with each one-unit increase in CTI corresponding to a 19% higher risk of stroke (23).

An increasing number of studies have begun to examine the potential association between CTI and CVD. However, evidence regarding long-term cumulative CTI (cumCTI) exposure is limited. The association between cumCTI and CVD risk—particularly in conjunction with depression—remains unclear. This study aims to investigate the combined influence of cumCTI and depression on the risk of CVD.

## 2. Methods

### 2.1 Study Population

The data were derived from the China Health and Retirement Longitudinal Study (CHARLS), a nationally representative survey designed to collect high-quality micro-level information on Chinese adults aged 45 years and older. CHARLS was initiated in 2011 and conducts follow-up surveys every two to three years. To date, five survey waves have been completed. Biomarker data were collected in Wave 1 (2011) and Wave 3 (2015). In the present study, Wave 3 was treated as the baseline survey, which covered 150 county-level units and 450 community/village units and included approximately 17,000 participants from about 10,000 households. Participants were subsequently followed through two follow-up waves: Wave 4 (2018) and Wave 5 (2020). The CHARLS study was approved by the Ethics Committee of Peking University, and written informed consent was obtained from all participants (IRB 00001052-11015). In this study, participants were excluded if they met any of the following criteria:

- Missing baseline data on CRP, triglyceride, fasting glucose, or depression-related variables.
- A confirmed diagnosis of CVD at baseline.
- Missing follow-up information on CVD across Waves 4–5.

**Figure 1:**
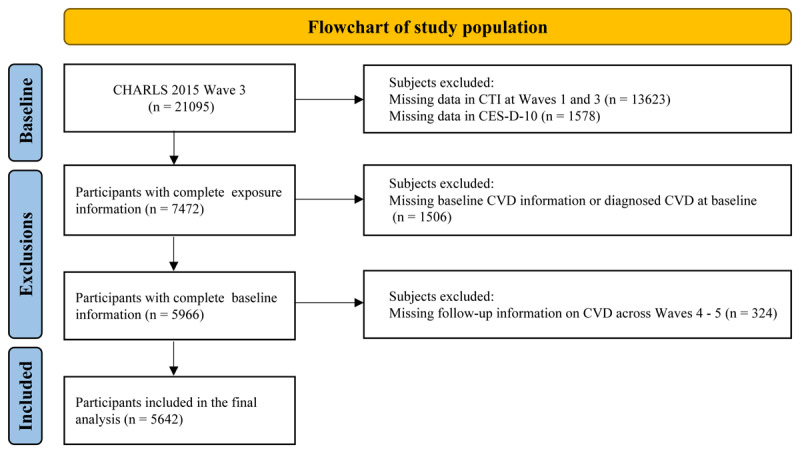
Flowchart of study population.

After applying these exclusion criteria, a total of 5,642 participants were included in the final analysis. Detailed procedures for data extraction from the cohorts are depicted in the flowcharts (Figure 1).

### 2.2 Data Assessment

#### 2.2.1 Exposure

CTI was calculated using the following formula: *CTI* = 0.412 × ln [*hs-CRP* (mg/L)] + ln [(*triglycerides* (mg/dL) × *fasting glucose* (mg/dL))/2]. The cumCTI was defined as the cumulative burden of CTI across Wave 1 and Wave 3 and was calculated as: (*CTI*_2011_ + *CTI*_2015_)/2 × *time*_1–2_, where CTI_2011_ and CTI_2015_ represent the CTI values at each visit, and time_1–2_ represents the interval between the two visits (20). Depression was assessed using the 10-item Center for Epidemiologic Studies Depression Scale (CES-D-10). Each of the 10 items is rated on a 4-point Likert scale of 0–3 points (Table S1). The total CES-D-10 score was calculated as the unweighted sum of the 10 individual items, yielding a possible score range of 0–30, with higher scores indicating greater depressive symptomatology. According to the widely adopted cut-off established in the original validation study, a score of 0–9 indicates no depression, whereas a score of ≥10 is interpreted as the presence of depression (24). A composite exposure variable was constructed by jointly categorizing the cumCTI level and depression. To facilitate interpretation of joint exposure effects, cumCTI was dichotomized at the median and combined with binary depression status to construct four exposure groups. This categorical approach yielded four relatively balanced and clinically interpretable groups (low cumCTI without depression, low cumCTI with depression, high cumCTI without depression, and high cumCTI with depression) and was primarily used to describe joint exposure patterns. Additionally, continuous cumCTI analyses were performed to assess exposure–response relationships and minimize potential information loss associated with categorization.

#### 2.2.2 Covariates

Covariates were grouped into three categories: sociodemographic factors, lifestyle behaviors, and health conditions. Sociodemographic variables included age (treated as a continuous variable in the main analysis and categorized as <60 or ≥60 years in subgroup analyses), gender, and marital status (married with spouse present vs. other), all of which were considered categorical variables. Lifestyle factors included smoking status (former or current vs. never) and drinking status (former or current vs. never), as well as night sleeping time and nap duration (both continuous). Health-related variables—hypertension, dyslipidemia, dysglycemia, kidney disease, and psychiatric problems—were all treated as categorical variables.

#### 2.2.3 Outcome

The primary outcome was incident CVD, defined according to CHARLS criteria as a composite endpoint encompassing heart disease (including myocardial infarction, coronary heart disease, angina, congestive heart failure, and other cardiac conditions) and stroke. Incident CVD events were identified by integrating information from two follow-up waves (Wave 4 and Wave 5). As exact dates of CVD event onset were not available in CHARLS, event time was approximated using survey wave–based information. Specifically, the time to event was defined as the interval from baseline to the first wave at which a CVD event was reported, corresponding to follow-up assessments conducted at approximately three and five years.

### 2.3 Data Collection

Blood samples were available only in Wave 1 (2011) and Wave 3 (2015). Exposure variables—including CRP, triglycerides, and fasting glucose—were obtained from these two survey waves. CES-D-10 scores and all covariates were derived from the Wave 3 baseline data. Outcome information was ascertained from Wave 4 (2018) and Wave 5 (2020).

### 2.4 Statistical Analysis

Continuous variables were summarized as means with standard deviations (SD) for approximately normally distributed data or as medians with interquartile ranges (IQR) for skewed distributions. Between-group differences were compared using the t-test or one-way analysis of variance (ANOVA) for normally distributed variables, and the Mann–Whitney U test or Kruskal–Wallis test for non-normally distributed variables. Categorical variables were presented as counts and percentages and were compared using the chi-square test.

Missing data on covariates were handled according to the extent of missingness. Variables with minimal missingness (≤10%) were imputed using the mode for categorical variables, the mean for approximately normally distributed continuous variables, and the median for skewed continuous variables. For variables with moderate missingness (10–20%), multiple imputation by chained equations was prespecified to account for uncertainty associated with missing data. Variables with substantial missingness (>20%) would be considered for potential exclusion because high missingness could compromise estimate stability, particularly when available evidence did not support their role as essential confounders in the exposure-outcome association. In the present study, the missingness rates of all included covariates are presented in Table S2. No variables were excluded due to missing data.

Cox proportional hazards regression was applied to estimate the joint association of cumCTI and depression with the risk of CVD. Four sequential models were constructed: Model 1 was the crude model, Model 2 adjusted for sociodemographic factors, Model 3 additionally adjusted for lifestyle behaviors, and Model 4 further adjusted for health conditions. The proportional hazards assumption was assessed using Schoenfeld residuals. Restricted cubic spline (RCS) regression with four knots was used to assess potential nonlinear associations between cumCTI and CVD risk. Nonlinearity was tested using Wald chi-square statistics for the spline terms, and RCS analyses were stratified by depression status to compare dose–response patterns. Interactions between cumCTI and depression were evaluated sequentially. Linear interaction between continuous cumCTI and categorical depression status was assessed on the multiplicative scale by including a product term in Cox models, with significance tested using likelihood ratio tests. Nonlinear interactions were further examined using RCS-based interaction models. Multiplicative interaction between categorical cumCTI and categorical depression status was also assessed. Finally, the incremental predictive value of the combined cumCTI-depression indicator beyond each individual component was evaluated using integrated discrimination improvement (IDI) and net reclassification improvement (NRI). Subgroup analyses were conducted across demographic, lifestyle, and health conditions, including age, gender, smoking status, drinking status, hypertension, and dyslipidemia. Hazard ratios (HR) and 95% confidence intervals (CI) within each subgroup were estimated using Cox models adjusted for the same covariates as in the primary analysis. The significance of heterogeneity was evaluated using interaction terms.

Sensitivity analyses were conducted to evaluate the robustness of the primary findings. First, continuous variables were winsorized to mitigate the influence of extreme values, with observations below the 1st percentile and above the 99th percentile replaced by the corresponding percentile thresholds. Second, to assess the impact of missing data handling, all covariates were imputed using multiple imputation with five imputations, and estimates were pooled according to Rubin’s rules. Third, to address potential temporal ambiguity between depression and cumCTI exposure, depressive symptom trajectories were constructed using CES-D-10 assessments from both 2011 and 2015. Participants were categorized into four groups: without depression, new-onset depression, remitted depression, and persistent depression. These trajectory categories were then jointly analyzed with cumCTI in Cox proportional hazards models. Fourth, to address potential interval censoring in event time ascertainment, events first reported at the 2018 follow-up were assigned the midpoint between the 2015 and 2018 waves, and those first reported at the 2020 follow-up were assigned the midpoint between the 2018 and 2020 waves. Fifth, additional sensitivity analyses were performed using tertile- and quartile-based categorizations of cumCTI to evaluate the robustness of the observed categorical associations. Sixth, cause-specific analyses were performed by separately evaluating heart disease and stroke outcomes to examine whether the associations differed across these pathophysiologically distinct entities. Seventh, survey-weighted Cox proportional hazards models were performed using individual sampling weights adjusted for household- and individual-level non-response. Eighth, we further adjusted for body-mass index (BMI), socioeconomic status, educational attainment, residence area, physical activity level, and the use of antihypertensive, lipid-lowering, and antidiabetic medications to evaluate the potential influence of residual confounding. BMI was included as a continuous variable and categorized as an anthropometric measure. Socioeconomic status was defined according to quartiles of individual assets. Educational attainment was categorized as low (< elementary school), middle (elementary or middle school), and high (≥ high school). Residence area was categorized as urban, peri-urban, and rural. Antihypertensive, lipid-lowering, and antidiabetic medications were modeled as binary variables (use vs. non-use). Participants without the corresponding disease were coded as non-users for analytical purposes. Physical activity level was estimated based on weekly activity frequency, activity duration, and metabolic equivalent of task (MET) values, and categorized into low, moderate, and high levels according to the International Physical Activity Questionnaire (IPAQ) classification criteria. Because physical activity data had a substantial proportion of missing values (>50%), multiple imputation was considered potentially unreliable for this variable. Therefore, a missing-indicator category was additionally introduced for physical activity level in the sensitivity analyses.

All statistical analyses were conducted using R software (version 4.5.2). A two-sided *p* < 0.05 was considered statistically significant.

## 3. Results

### 3.1 Baseline Characteristics of the Study Population

A total of 5,642 participants were included in the analysis: 909 with high cumCTI and depression, 1,912 with high cumCTI without depression, 950 with low cumCTI and depression, and 1,871 with low cumCTI without depression. Compared with participants without depression, those with depression were more likely to be female, less likely to live with a spouse, and had shorter nocturnal sleep duration and nap duration. Participants with high cumCTI had a higher prevalence of hypertension, dyslipidemia, dysglycemia, and kidney disease. Detailed baseline characteristics are presented in Table 1.

**Table 1:** Baseline Characteristics of Participants Stratified by Cumulative CTI and Depression.


VARIABLES	OVERALL N = 5,642^1^	HIGH cumCTI WITH DEPRESSION N = 909^1^	HIGH cumCTI WITHOUT DEPRESSION N = 1,912^1^	LOW cumCTI WITH DEPRESSION N = 950^1^	LOW cumCTI WITHOUT DEPRESSION N = 1,871^1^	*p*-VALUE^2^

Age	71 ± 9	72 ± 9	72 ± 9	71 ± 8	72 ± 9	0.045

Gender						<0.001

male	2,574 (46%)	292 (32%)	917 (48%)	367 (39%)	998 (53%)	

female	3,068 (54%)	617 (68%)	995 (52%)	583 (61%)	873 (47%)	

Marital status						<0.001

Married with spouse present	4,711 (83%)	718 (79%)	1,640 (86%)	762 (80%)	1,591 (85%)	

Other	931 (17%)	191 (21%)	272 (14%)	188 (20%)	280 (15%)	

Smoke						<0.001

No	3,253 (58%)	592 (65%)	1,068 (56%)	590 (62%)	1,003 (54%)	

Yes	2,389 (42%)	317 (35%)	844 (44%)	360 (38%)	868 (46%)	

Drink						<0.001

No	3,667 (65%)	654 (72%)	1,238 (65%)	626 (66%)	1,149 (61%)	

Yes	1,975 (35%)	255 (28%)	674 (35%)	324 (34%)	722 (39%)	

Night sleeping time (hours)	6.46 ± 1.95	5.96 ± 2.15	6.77 ± 1.82	5.84 ± 2.07	6.70 ± 1.78	<0.001

Nap time (minutes)	39 ± 46	38 ± 45	43 ± 47	33 ± 44	38 ± 46	<0.001

Hypertension						<0.001

No	4,200 (74%)	590 (65%)	1,313 (69%)	769 (81%)	1,528 (82%)	

Yes	1,442 (26%)	319 (35%)	599 (31%)	181 (19%)	343 (18%)	

Dyslipidemia						<0.001

No	5,117 (91%)	782 (86%)	1,670 (87%)	895 (94%)	1,770 (95%)	

Yes	525 (9.3%)	127 (14%)	242 (13%)	55 (5.8%)	101 (5.4%)	

Dysglycemia						<0.001

No	5,298 (94%)	801 (88%)	1,735 (91%)	924 (97%)	1,838 (98%)	

Yes	344 (6.1%)	108 (12%)	177 (9.3%)	26 (2.7%)	33 (1.8%)	

Kidney disease						<0.001

No	5,404 (96%)	856 (94%)	1,839 (96%)	892 (94%)	1,817 (97%)	

Yes	238 (4.2%)	53 (5.8%)	73 (3.8%)	58 (6.1%)	54 (2.9%)	

Psychiatric problems						0.022

No	5,599 (99%)	899 (99%)	1,906 (100%)	938 (99%)	1,856 (99%)	

Yes	43 (0.8%)	10 (1.1%)	6 (0.3%)	12 (1.3%)	15 (0.8%)	

Glycated Hemoglobin (%)	5.97 ± 0.98	6.25 ± 1.40	6.20 ± 1.18	5.71 ± 0.50	5.74 ± 0.47	<0.001

Glucose (mg/dl)	103 ± 33	113 ± 44	113 ± 42	92 ± 15	94 ± 14	<0.001

Uric Acid (mg/dl)	4.90 ± 1.39	5.04 ± 1.41	5.25 ± 1.41	4.40 ± 1.22	4.74 ± 1.35	<0.001

Total Cholesterol (mg/dl)	185 ± 36	194 ± 41	191 ± 39	177 ± 32	179 ± 31	<0.001

LDL Cholesterol (mg/dl)	103 ± 29	105 ± 30	104 ± 31	99 ± 27	102 ± 26	<0.001

HDL Cholesterol (mg/dl)	52 ± 12	49 ± 10	48 ± 10	56 ± 13	55 ± 12	<0.001

Creatinine (mg/dl)	0.80 ± 0.26	0.77 ± 0.23	0.81 ± 0.25	0.79 ± 0.39	0.80 ± 0.21	<0.001

cumCTI	38.94 ± 3.04	41.37 ± 2.29	41.31 ± 2.18	36.44 ± 1.47	36.60 ± 1.42	<0.001

Total depression score	8 ± 6	15 ± 5	4 ± 3	15 ± 5	4 ± 3	<0.001

Event of CVD						<0.001

No event	4,539 (80%)	653 (72%)	1,526 (80%)	739 (78%)	1,621 (87%)	

Event	1,103 (20%)	256 (28%)	386 (20%)	211 (22%)	250 (13%)	


^1^Mean ± SD; n (%). ^2^Kruskal-Wallis rank sum test; Pearson’s Chi-squared test. cumCTI: cumulative C-reactive protein–triglyceride–glucose index, HDL: High-Density Lipoprotein, LDL: Low-Density Lipoprotein, CVD: cardiovascular disease.

### 3.2 Joint Association of cumCTI and Depression with CVD Risk

**Figure 2:**
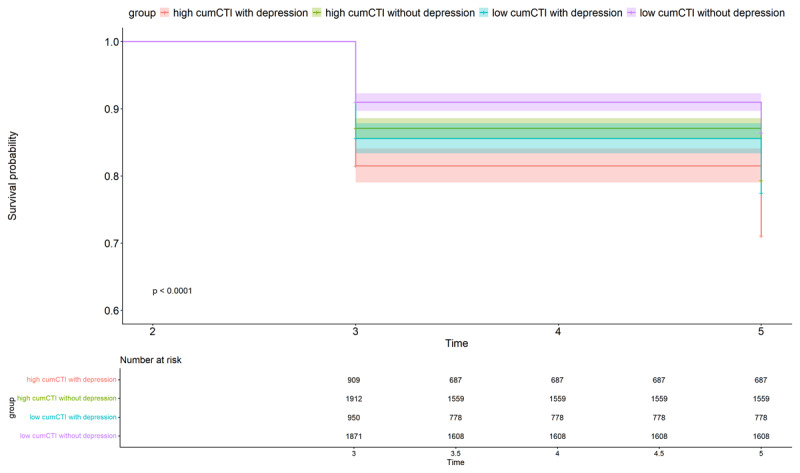
Kaplan–Meier curves for survival probability across cumCTI-depression categories.

Kaplan–Meier curves of survival probability across cumCTI–depression categories showed clear separation of risk trajectories, with the lowest survival probability observed in participants with high cumCTI and depression (Figure 2). Cox proportional hazards models were applied, with sequential adjustment for sociodemographic, lifestyle, and clinical covariates. Compared with participants with low cumCTI without depression, all combined exposure categories were associated with a higher risk of CVD across all adjusted models. The proportional hazards assumption was evaluated using Schoenfeld residuals, and no evidence of violation was observed (all *p* > 0.05) (Table S3).

In the crude model, CVD risk was higher in participants with low cumCTI and depression compared with the reference group (HR 1.74, 95% CI 1.45–2.09, *p* < 0.001). Among individuals without depression, high cumCTI was associated with increased risk (HR 1.57, 95% CI 1.34–1.84, *p* < 0.001). The highest risk was observed in those with both high cumCTI and depression (HR 2.31, 95% CI 1.94–2.75, *p* < 0.001). After sequential adjustment for sociodemographic factors (Model 2, Figure S1) and lifestyle characteristics (Model 3, Figure S2), these associations remained evident. In the fully adjusted Model 4 (Figure S3), which additionally controlled for chronic disease, the associations persisted. Low cumCTI with depression remained significantly associated with an increased risk of CVD (HR = 1.74, 95% CI 1.44–2.09, *p* < 0.001). Among participants with high cumCTI, a clear risk gradient was observed, with an HR of 1.35 (95% CI 1.15–1.59, *p* < 0.001) for those without depression and 1.83 (95% CI 1.53–2.20; *p* < 0.001) for those with depression (Table 2). Overall, a clear graded association was evident, with CVD risk increasing across cumCTI categories and depression status, and the combined exposure of high cumCTI and depression consistently showed the highest risk across all models.

**Table 2:** Cox proportional hazards models of the joint association of cumulative CTI and depression with CVD risk.


GROUP	MODEL 1		MODEL 2		MODEL 3		MODEL 4	
			
	HR (95%CI)	*p*	HR (95%CI)	*p*	HR (95%CI)	*p*	HR (95%CI)	*p*

Low cumCTI without depression	ref	ref	ref	ref	ref	ref	ref	ref

Low cumCTI with depression	1.74 (1.45–2.09)	<0.001	1.79 (1.49–2.15)	<0.001	1.75 (1.45–2.11)	<0.001	1.74 (1.44–2.09)	<0.001

High cumCTI without depression	1.57 (1.34–1.84)	<0.001	1.58 (1.35–1.85)	<0.001	1.56 (1.33–1.83)	<0.001	1.35 (1.15–1.59)	<0.001

High cumCTI with depression	2.31 (1.94–2.75)	<0.001	2.28 (1.91–2.72)	<0.001	2.21 (1.85–2.64)	<0.001	1.83 (1.53–2.20)	<0.001


cumCTI: cumulative C-reactive protein–triglyceride glucose index, CVD: cardiovascular diseases, HR: hazard ratio, CI: confidence interval. Model 1: unadjusted. Model 2: adjusted for age, gender, and marital status. Model 3: further adjusted for smoke, drink, night sleeping time, and nap time. Model 4: further adjusted for hypertension, dyslipidemia, dysglycemia, kidney disease, and psychiatric problems.

To assess the robustness of the exposure–outcome association beyond categorical classification, cumCTI was additionally modeled as a continuous variable. In the overall population, cumCTI was consistently associated with an increased risk of CVD, with minimal attenuation after sequential adjustment. Similar associations were observed among participants with and without depression, with consistent effect estimates across models. In the fully adjusted model, each 1-unit increase in cumCTI was associated with a higher risk of CVD in the overall population (HR 1.035, 95% CI 1.015–1.056, *p* < 0.001), as well as among participants with depression (HR 1.033, 95% CI 1.012–1.054, *p* = 0.014) and without depression (HR 1.043, 95% CI 1.016–1.071, *p* = 0.001) (Table S4).

**Figure 3:**
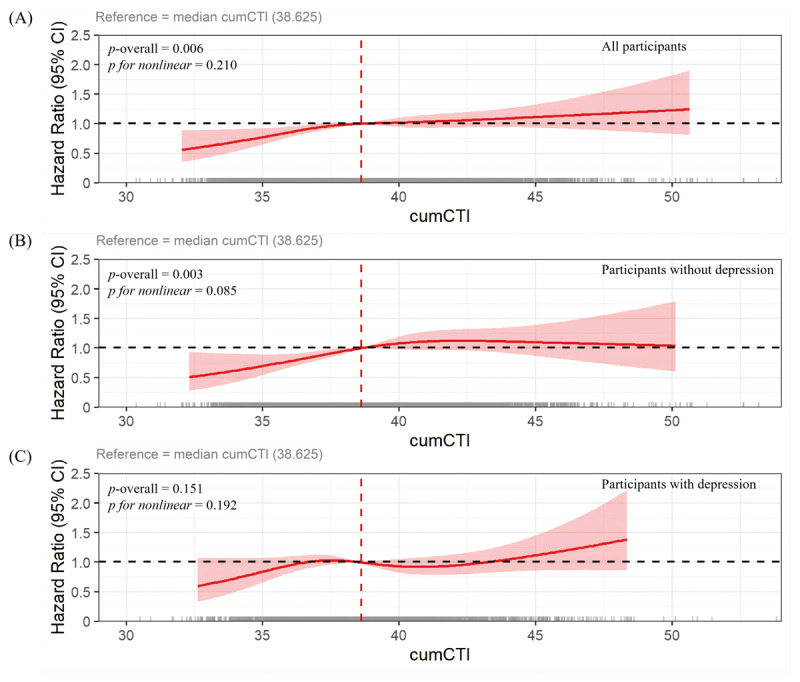
The RCS analysis between the cumulative CTI and CVD incidence. **(A)** all participants; **(B)** participants without depression; **(C)** participants with depression.

RCS analyses demonstrated a linear association between cumCTI and CVD risk in the overall population, with no evidence of nonlinearity (*p* for overall = 0.006; *p* for nonlinearity = 0.210). Among participants without depression, the association remained nonsignificant for nonlinearity (*p* for nonlinearity = 0.085), suggesting no clear deviation from linearity. In participants with depression, neither the overall association (*p* for overall = 0.151) nor the nonlinear component (*p* for nonlinearity = 0.192) was statistically significant (Figure 3).

### 3.3 Interaction Analysis

No significant linear interaction was observed between continuous cumCTI and depression status (*p* for interaction = 0.221, Table S5). In the RCS-based model, a marginal nonlinear interaction between cumCTI and depression status was detected (χ*²* = 6.86, *df* = 3, *p* for interaction = 0.076, Table S6), suggesting potential differences in the shape of the cumCTI-CVD association across depression status. In contrast, a statistically significant multiplicative interaction was observed between categorized cumCTI levels and depression status (χ*²* = 3.93, *df* = 1, *p* for interaction = 0.047) (Table S7), indicating possible effect modification of the cumCTI–CVD association by depression status.

### 3.4 Predictive Improvement of the cumCTI-Depression

To evaluate whether combining cumCTI with depression improved the prediction of CVD, the predictive performance of the composite indicator was compared with that of the individual components (cumCTI or depression alone) using the IDI and NRI. Compared with cumCTI alone, the composite measure provided additional predictive value, as reflected by a significant improvement in discrimination (IDI = 0.0078, 95% CI 0.0032–0.0136; *p* < 0.001) and a positive overall NRI of 0.232. Similarly, relative to depression status alone, the combined indicator resulted in a significant increase in discrimination (IDI = 0.0019, 95% CI 0.0004–0.0050; *p* < 0.001) and favorable net reclassification (NRI = 0.128). Overall, these findings suggest that the combined assessment of cumCTI and depression may modestly improve CVD risk stratification beyond either component alone (Table S8).

### 3.5 Subgroup Analyses

Subgroup analyses were conducted across demographic, lifestyle, and health conditions, including age, gender, smoking status, drinking status, hypertension, and dyslipidemia. Overall, the associations between cumCTI-depression and CVD risk were generally consistent across subgroups. No significant interaction was detected (all *p* for interaction >0.05), indicating that the joint effect of cumCTI and depression did not differ materially across these subgroups (Table S9).

### 3.6 Sensitivity Analysis

Sensitivity analyses were conducted to assess the robustness of the primary findings. First, continuous variables were winsorized to reduce the influence of extreme values, and the re-estimated models produced results consistent with the primary analysis (Table S10). Second, missing covariate data were addressed using multiple imputation with five imputations, and estimates were pooled according to Rubin’s rules (Table S11). Third, in an additional sensitivity analysis using depressive symptom trajectories, the associations between joint exposure categories and CVD remained statistically significant with a graded effect after sequential adjustment. A possible effect modification by depression trajectory was also observed (*p* = 0.03) (Table S12). Fourth, in the sensitivity analysis accounting for potential interval censoring using midpoint imputation, the results were consistent with those of the primary analysis, with no material changes in effect estimates or statistical significance (Table S13). Fifth, in sensitivity analyses using tertile- and quartile-based categorizations of cumCTI, no statistically significant effect modification between categorized cumCTI and depression status was observed (*p* = 0.16 for tertiles and 0.15 for quartiles). Sixth, additional analyses for specific outcomes showed that the cumCTI-depression were consistently associated with elevated risks of both heart disease (Table S14) and stroke (Table S15). The associations appeared stronger for stroke, while a possible effect modification by depression was observed only for heart disease (*p* = 0.01). Seventh, survey-weighted analyses yielded effect estimates broadly consistent with the primary findings (Table S16). However, the possible effect modification by depression was attenuated and no longer statistically significant after incorporating survey weights (*p* = 0.20). Eighth, the sensitivity analysis, additionally adjusting for BMI, socioeconomic status, educational attainment, residence area, physical activity level, and antihypertensive, lipid-lowering, and antidiabetic medications, yielded results broadly consistent with the primary analysis (Table S17). In the fully adjusted model, evidence of possible effect modification by depression status was still observed (*p* = 0.02).

## 4. Discussion

In this cohort of 5,642 participants followed for three to five years, the combined cumCTI–depression indicator was strongly associated with CVD risk, exhibiting a clear graded association. These associations remained robust after sequential adjustment for demographic, lifestyle, and clinical covariates. In addition, stratified categorical analyses provided supportive evidence of possible effect modification by depression. Subgroup analyses indicated that the associations were generally consistent across strata of age, sex, smoking status, alcohol consumption, hypertension, and dyslipidemia. Furthermore, IDI and NRI analyses demonstrated that the combined cumCTI–depression indicator improved risk discrimination and reclassification beyond either component alone, highlighting its potential clinical utility. Overall, integrating cumCTI with depression may facilitate earlier identification of individuals at elevated cardiovascular risk and inform more targeted preventive strategies.

As one of the leading global public health challenges, depression has shown a steadily increasing prevalence and disease burden worldwide, and is commonly comorbid with CVD (25). Prior evidence indicates that depressive symptoms—even below the diagnostic threshold—are significantly associated with incident CVD (8). Jian et al. combined the modified TyG index with depression and reported that the newly constructed composite indicator was positively associated with stroke risk, with a significant positive interaction (26). Similarly, Tian et al. measured systemic inflammation using CRP and found that elevated inflammatory levels may amplify the CVD risk associated with depression (27). Several biological mechanisms may underlie these associations. Individuals with depression often experience chronic and sustained psychological stress, leading to persistent activation of the hypothalamic–pituitary–adrenal (HPA) axis and elevated circulating cortisol (28). Increased cortisol levels, in turn, can promote CVD via multiple pathways, including insulin resistance, hypertension, and dyslipidemia (29,30,31). Moreover, inflammation represents another critical mechanistic pathway. Depression has been shown to exhibit dose–response relationships with multiple classical inflammatory biomarkers, such as CRP, interleukin-1 (IL-1), and interleukin-6 (IL-6) (32), and the two may mutually reinforce each other, further amplifying their detrimental effects (33). Although prior studies have explored composite metabolic-psychological or inflammatory-psychological indicators for CVD risk assessment, they have not integrated systemic inflammation, metabolic status, and psychological distress into a unified framework, nor captured the cumulative metabolic–inflammatory burden over time.

The CTI, proposed by Ruan et al. in 2022 as a composite biomarker integrating metabolic and inflammatory status, has gained increasing attention. Initially developed to predict cancer outcomes, it has since been validated across diverse populations and disease contexts. Evidence from the Jiangxi-ADHF II cohort showed that elevated CTI levels were strongly associated with short-term mortality in acute decompensated heart failure patients (34). Analyses from the National Health and Nutrition Examination Survey (NHANES) further demonstrated significant positive associations between CTI and CVD mortality, total CVD incidence, and all-cause mortality (21,35), while CHARLS data indicated that each unit increase in CTI corresponded to an 11% higher CVD risk (36). Beyond epidemiological links, CTI exhibits superior predictive performance relative to conventional metabolic or inflammatory markers. Among patients treated with percutaneous coronary intervention, CTI outperformed TyG and other related indicators in predicting recurrent major adverse cardiac and cerebrovascular events and significantly enhanced model discrimination (37). Consistent findings from NHANES showed an 18% increase in CVD odds per unit increase in CTI, with analysis highlighting greater contributions of CTI than CRP or TyG to CVD classification (38). A public-database study with external validation also identified CTI as an independent predictor of all-cause and premature mortality, outperforming TyG (39). CTI-derived indicators further support its clinical relevance; CHARLS data revealed that persistently high CTI levels markedly increased stroke risk among non-diabetic adults (40), and cumCTI and CTI trajectories were strongly related to stroke risk, with consistently high (HR = 1.53) or progressively rising CTI (HR = 1.75) indicating substantially higher risk (41). Given its multidimensional nature, several CTI-based composite indicators have been proposed. Joint assessment of CTI and adiposity showed synergistic effects on CVD risk (42), and integrating CTI with the C-reactive protein–Albumin–Lymphocyte index improved discrimination for new-onset coronary heart disease (43). Notably, higher CTI has also been linked to depressive and anxiety symptoms (44,45), suggesting potential value in combining CTI with depression status to refine psycho–cardiometabolic risk stratification.

This study has several strengths; it is the first investigation to examine the joint effects of cumCTI and depression on CVD events, thereby providing insights into differential CVD risk by depression status. The study utilized objectively measurable biomarkers (CTI) together with a standardized psychological assessment tool (CES-D-10), all of which are cost-effective and easy to collect. These features highlight the potential for clinical translation and future implementation. However, several limitations should be acknowledged. First, CVD events were determined based on self-reported prior physician diagnoses rather than verified clinical records, which may therefore be subject to recall bias and potential misclassification. Participants with depressive symptoms may differ in healthcare utilization, symptom perception, memory recall, or disease reporting behavior, which could increase the likelihood of reporting physician-diagnosed cardiovascular conditions. Therefore, differential misclassification of self-reported CVD outcomes across depression status cannot be excluded. Nevertheless, self-reported CVD has shown acceptable validity in several large population-based cohort studies (46,47). CHARLS-specific evidence also supports the general reliability of self-reported chronic disease data. Ning et al. compared self-reported hypertension and diabetes with biomedical measurements within the CHARLS cohort and reported high specificity (96.3% for hypertension and 98.3% for diabetes), moderate sensitivity (56.3% and 61.5%, respectively), and moderate agreement (κ = 0.57–0.65). Although this validation study did not directly evaluate CVD outcomes, it is relevant because CHARLS uses the same physician-diagnosis self-report format for chronic conditions, including heart disease and stroke (48). Second, the CES-D-10, a shortened version of the original CES-D scale, may not comprehensively capture the full spectrum of depressive symptoms, and its narrower scoring range may limit its sensitivity in distinguishing depression status. Third, the CHARLS survey did not systematically capture certain CVD conditions, such as peripheral artery disease, which are clinically important but often less perceptible to participants. This may result in incomplete identification of overall CVD events and, consequently, an underestimation of the true CVD risk level. Further clinical studies with more comprehensive and detailed data are warranted.

## 5. Conclusion

This study demonstrated that the combined cumCTI-depression indicator was associated with CVD risk, exhibiting a clear graded association that remained robust after sequential adjustment for demographic, lifestyle, and clinical covariates. Categorical analyses suggested possible effect modification by depression status. Furthermore, the joint indicator improved risk discrimination and reclassification beyond either component alone. Overall, integrating cumCTI with depression status may contribute to improved cardiovascular risk stratification and support future research on targeted prevention approaches.

## Additional File

The additional file for this article can be found as follows:

10.5334/gh.1565.s1 Supplementary Material. Figures S1–S3 and Tables S1–S17.

## Data Availability

The dataset used in this study was publicly available and can be accessed at https://charls.pku.edu.cn/en. The data supporting the findings of this study are available from the corresponding author upon reasonable request.

## Abbreviations

CES-D-10: Center for Epidemiologic Studies Depression Scale-10

CHARLS: China Health and Retirement Longitudinal Study

CRP: C-reactive protein

CTI: C-reactive protein–triglyceride–glucose index

cumCTI: cumulative C-reactive protein–triglyceride–glucose index

CVD: cardiovascular disease

IQR: interquartile ranges

NHANES: National Health and Nutrition Examination Survey

TyG: triglyceride–glucose

RCS: restricted cubic spline

BMI: body mass index

IDI: integrated discrimination improvement

NRI: net reclassification improvement

MET: metabolic equivalent of task

IPAQ: International Physical Activity Questionnaire
