# Supplementary material for: Joint Associations of Cumulative C-Reactive Protein–Triglyceride–Glucose Index and Depression with Cardiovascular Outcomes in Middle-Aged and Older Adults: A National Prospective Cohort Study
Source: Glob Heart. 2026 Jun 17;21(1):47. doi: 10.5334/gh.1565 (PMC13281729; doi:10.5334/gh.1565)
Supplement: Supplementary Material. — Figures S1–S3 and Tables S1–S17. [file gh-21-1-1565-s1.pdf]

Supplementary Material

Joint Associations of CTI and Depression Severity with Cardiovascular Outcomes in Middle-Aged and Older Adults: A Nationally Prospective Cohort Study

Contents

1. Figure S1. Forest plot for cumCTI-depression and CVD risk adjusted for sociodemographic factors…………….……...……………………...……3
2. Figure S2. Forest plot for cumCTI-depression and CVD risk adjusted for sociodemographic factors and lifestyle behaviors. ……………….…………….…4
3. Figure S3. Forest plot for cumCTI-depression and CVD risk adjusted for sociodemographic factors, lifestyle behaviors and health conditions.…………..……5
4. Table S1. The 10-item Center for Epidemiologic Studies Depression Scale……..6
5. Table S2. Summary of missing value proportions for covariates……………...……7
6. Table S3. Assessment of the Cox proportional hazards assumption………………8
7. Table S4. Sensitivity analysis of the associations between cumulative CTI (continuous) and CVD risk across different populations…………………….…9
8. Table S5. Linear interaction analysis for cumCTI-depression in CVD risk………10
9. Table S6. Non-linear interaction analysis for cumCTI-depression in CVD risk…11
10. Table S7. Multiplicative interaction analysis for cumCTI-depression in CVD risk…………………………………………………………………………...…12
11. Table S8. Predictive improvement of the cumCTI-Depression for CVD risk……13
12. Table S9. Subgroup analysis for cumCTI-depression and CVD risk…………..……14
13. Table S10. Table S10. Sensitivity analysis for cumCTI-depression and CVD risk with 1%-99% winsorization.………………………………………………...…15
14. Table S11. Sensitivity analysis for cumCTI-depression and CVD risk with multiple imputation……………………………………………………………………..16
15. Table S12. Cox proportional hazards models of the associations between joint exposure categories and CVD risk using depressive symptom trajectories…..…17
16. Table S13. Cox proportional hazards models of the association between cumCTI-

depression categories and CVD risk using midpoint-imputed event times……...18

17. Table S14. Cox proportional hazards models of the association between cumCTI-depression categories and heart disease risk……………………………………..….19

18. Table S15. Cox proportional hazards models of the association between cumCTI-depression categories and stroke risk…………………………….………….....….20

19. Table S16. Survey-weighted Cox proportional hazards models of the association between cumCTI-depression categories and CVD risk……………………...…..…21

20. Table S17. Cox proportional hazards models of the association between cumCTI-depression categories and CVD risk with additional covariates……………..….22

Figure S1. Forest plot for cumCTI-depression and CVD risk adjusted for sociodemographic factors.

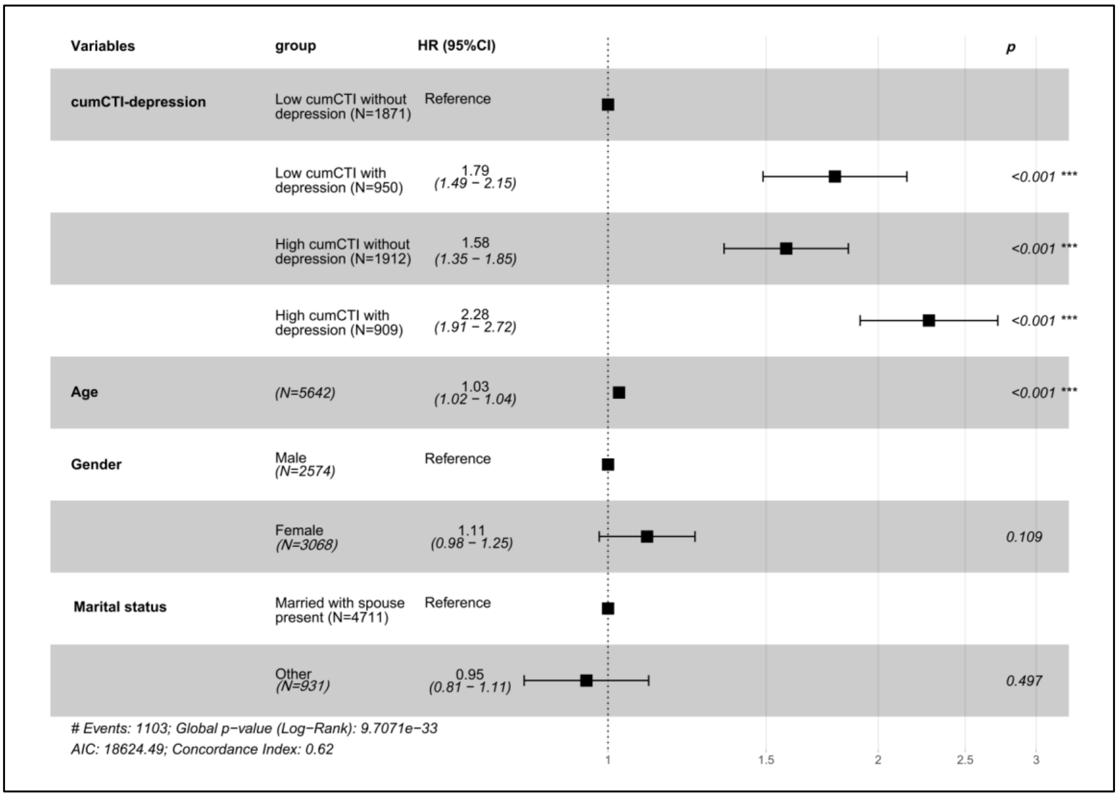

Figure S1. Forest plot for cumCTI-depression and CVD risk adjusted for sociodemographic factors. cumCTI: cumulative C-reactive protein–triglyceride–glucose index, CVD: cardiovascular diseases, HR: hazard ratio, CI: confidence interval.

Figure S2. Forest plot for cumCTI-depression and CVD risk adjusted for sociodemographic factors and lifestyle behaviors.

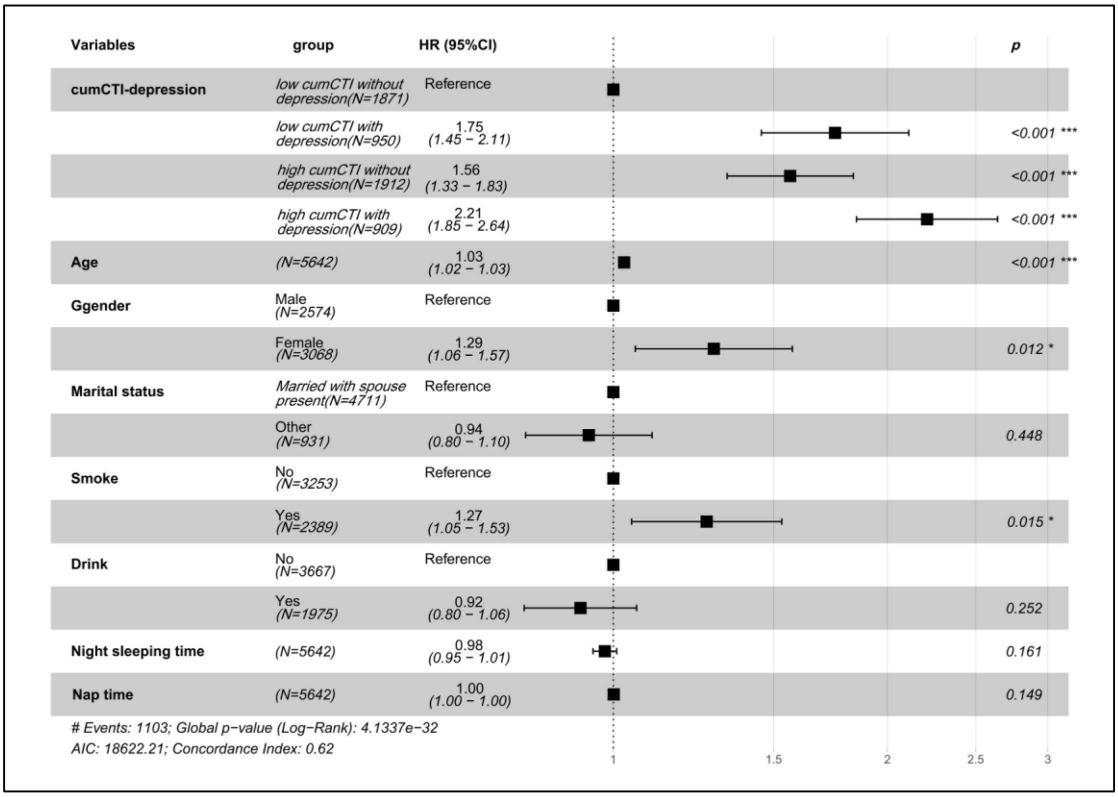

Figure S2. Forest plot for cumCTI-depression and CVD risk adjusted for sociodemographic factors and lifestyle behaviors. cumCTI: cumulative C-reactive protein–triglyceride–glucose index, CVD: cardiovascular diseases, HR: hazard ratio, CI: confidence interval.

Figure S3. Forest plot for cumCTI-depression and CVD risk adjusted for sociodemographic factors, lifestyle behaviors and health conditions.

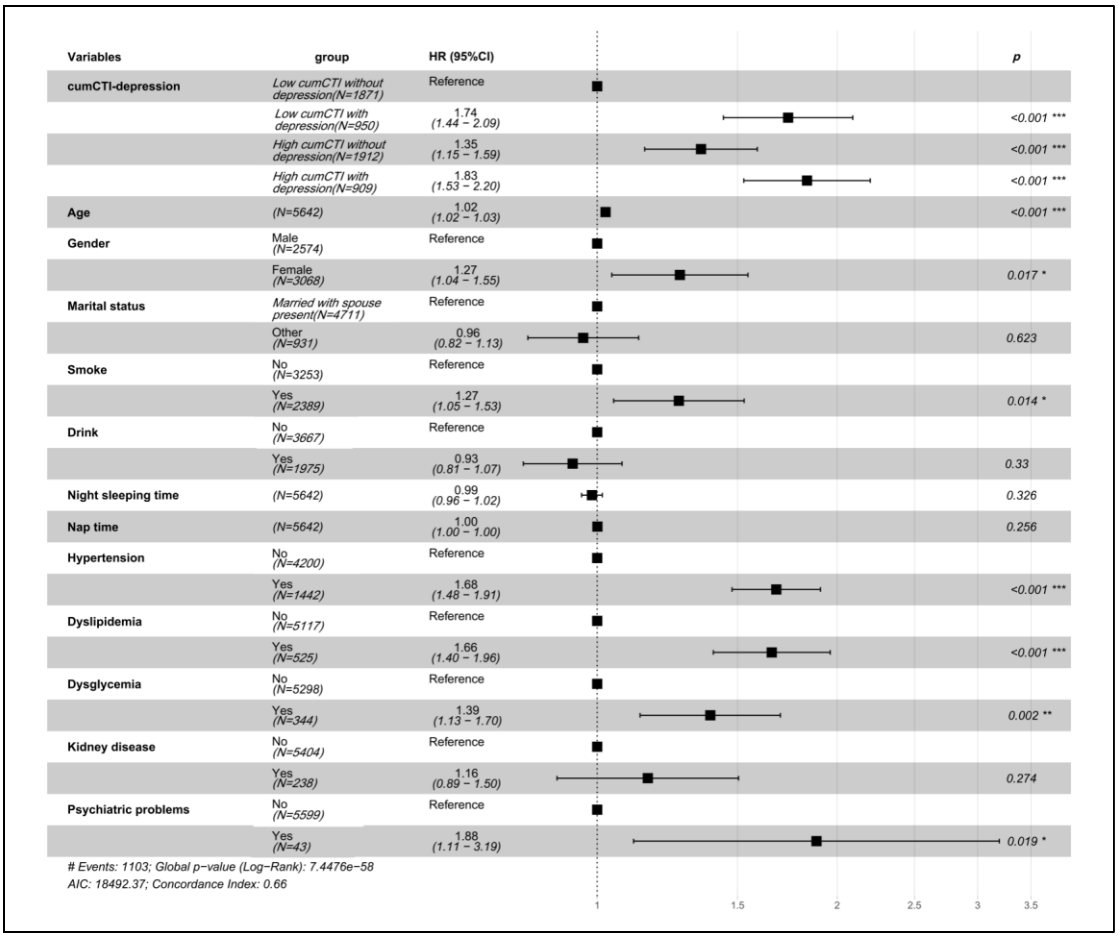

Figure S3. Forest plot for cumCTI-depression and CVD risk adjusted for sociodemographic factors, lifestyle behaviors and health conditions. cumCTI: cumulative C-reactive protein–triglyceride–glucose index, CVD: cardiovascular diseases, HR: hazard ratio, CI: confidence interval.

Table S1. The 10-item Center for Epidemiologic Studies Depression Scale.

Item	Response
(1) I was bothered by things that don't usually bother me.<br>(2) I had trouble keeping my mind on what I was doing.<br>(3) I felt depressed.<br>(4) I felt everything I did was an effort.<br>(5) I felt hopeful about the future.<br>(6) I felt fearful.<br>(7) My sleep was restless.<br>(8) I was happy.<br>(9) I felt lonely.<br>(10) I could not get "going."	(1) Rarely or none of the time (< 1 day)<br>(2) Some or a little of the time (1-2 days)<br>(3) Occasionally or a moderate amount of the time (3-4 days)<br>(4) Most or all of the time (5-7 days)

Table S2. Summary of missing value proportions for covariates.

ID	Proportion
Gender	0.00%
Birth Year	2.02%
Marital Status	0.00%
Night Sleeping Time	2.82%
Nap Time	1.86%
Smoke	0.00%
Drink	0.05%
Hypertension	4.34%
Dyslipidemia	3.95%
Dysglycemia	1.81%
Kidney Disease	1.40%
Mental Disorder	0.66%

Table S3. Assessment of the Cox proportional hazards assumption.

**Characteristics**	**Chi-squared Statistic**	**Degrees of freedom**	***p*-value of Chi-squared Statistic**
cumCTI-depression	2.131	3	0.546
Age	0.000	1	0.986
Gender	0.087	1	0.767
Marital status	0.123	1	0.726
Smoke	0.082	1	0.775
Drink	0.030	1	0.862
Night sleeping time	0.001	1	0.974
Nap time	1.462	1	0.227
Hypertension	0.704	1	0.402
Dyslipidemia	1.046	1	0.306
Dysglycemia	1.451	1	0.228
Kidney disease	0.013	1	0.910
Psychiatric problems	1.920	1	0.166
Global	9.944	15	0.823

cumCTI: cumulative C-reactive protein–triglyceride glucose index

Table S4. Cox proportional hazards models of the associations between cumulative CTI (continuous) and CVD risk across different populations

	**Model 1**		**Model 2**		**Model 3**		**Model 4**	
**Group**	HR (95%CI)	*p*	HR (95%CI)	*p*	HR (95%CI)	*p*	HR (95%CI)	*p*
Total	1.071(1.051–1.090)	<0.001	1.069 (1.050–1.089)	<0.001	1.068 (1.048–1.088)	<0.001	1.039 (1.019–1.060)	<0.001
Depression	1.060 (1.031–1.090)	<0.001	1.056 (1.026–1.086)	<0.001	1.055 (1.025–1.085)	<0.001	1.033 (1.012–1.054)	0.014
Non depression	1.079 (1.053–1.105)	<0.001	1.079 (1.053–1.106)	<0.001	1.078 (1.052–1.104)	<0.001	1.043 (1.016–1.071)	0.001

cumCTI: cumulative C-reactive protein–triglyceride glucose index, CVD: cardiovascular diseases, HR: hazard ratio, CI: confidence interval.

Model 1: unadjusted

Model 2: adjusted for age, gender, marital status

Model 3: further adjusted for smoke, drink, night sleeping time and nap time

Model 4: further adjusted for hypertension, dyslipidemia, dysglycemia, kidney disease, and psychiatric problems

Table S5. Linear interaction analysis for cumCTI-depression in CVD risk.

**Characteristics**	**Log-likelihood**	**Chi-squared Statistic**	**Degrees of freedom**	***p*-value of Chi-squared Statistic**
NULL	-9388.1			
cumCTI	-9363.8	48.6828	1	<0.001
Depression	-9336.2	55.1215	1	<0.001
Age	-9302.8	66.7248	1	<0.001
Gender	-9301.9	1.9459	1	0.163
Marital status	-9301.7	0.3917	1	0.531
Smoke	-9298.9	5.4784	1	0.019
Drink	-9298.4	1.1177	1	0.290
Night sleeping time	-9297.5	1.7197	1	0.190
Nap time	-9296.4	2.1933	1	0.139
Hypertension	-9256.3	80.2529	1	<0.001
Dyslipidemia	-9238.6	35.4234	1	<0.001
Dysglycemia	-9235.0	7.1990	1	<0.001
Kidney disease	-9234.3	1.3143	1	0.252
Psychiatric problems	-9232.2	4.2201	1	0.040
cumCTI-depression	-9231.5	1.4988	1	0.221

cumCTI: cumulative C-reactive protein–triglyceride glucose index, CVD: cardiovascular diseases.

Table S6. Non-linear interaction analysis for cumCTI-depression in CVD risk.

**Characteristics**	**Log-likelihood**	**Chi-squared Statistic**	**Degrees of freedom**	***p*-value of Chi-squared Statistic**
NULL	-9388.1			
Depression	-9361.7	52.7490	1	<0.001
RCS(cumCTI, 4)	-9334.4	54.5930	3	<0.001
Age	-9301.6	65.6330	1	<0.001
Gender	-9300.7	1.8404	1	0.176
Marital status	-9300.5	0.4457	1	0.504
Smoke	-9297.8	5.3481	1	0.021
Drink	-9297.2	1.1079	1	0.293
Night sleeping time	-9296.3	1.7819	1	0.182
Nap time	-9295.3	2.1272	1	0.145
Hypertension	-9255.1	80.4204	1	<0.001
Dyslipidemia	-9237.2	35.8090	1	<0.001
Dysglycemia	-9233.2	8.0050	1	0.005
Psychiatric problems	-9231.0	4.4094	1	0.036
Depression-RCS(cumCTI, 4)	-9227.5	6.8644	3	0.076

cumCTI: cumulative C-reactive protein–triglyceride glucose index, CVD: cardiovascular diseases, RCS: restricted cubic spline.

Table S7. Multiplicative interaction analysis for cumCTI level and depression in CVD risk.

**Characteristics**	**Log-likelihood**	**Chi-squared Statistic**	**Degrees of freedom**	***p*-value of Chi-squared Statistic**
Model 1	-9231.2			
Model 2	-9233.2	3.9288	1	0.047

cumCTI: cumulative C-reactive protein–triglyceride glucose index, CVD: cardiovascular diseases.

Model 1: cumCTI level * depression stage + age + gender + marital status + smoke + drink + night sleeping time + nap time + hypertension + dyslipidemia + dysglycemia + kidney disease + psychiatric problems

Model 2: cumCTI level + depression stage + age + gender + marital status + smoke + drink + night sleeping time + nap time + hypertension + dyslipidemia + dysglycemia + kidney disease + psychiatric problems

Table S8. Predictive improvement of the cumCTI-depression for CVD risk.

**Comparison**	**IDI**			**NRI**		
	IDI	95%CI	*p*-value	Overall	event	nonevent
Compared with CTI	0.0078	0.0032, 0.0136	< 0.001	0.2320	0.0644	0.2963
Compared with depression	0.0019	0.0004, 0.0050	< 0.001	0.1280	0.0626	0.0654

cumCTI: cumulative C-reactive protein–triglyceride glucose index, CVD: cardiovascular diseases, CI: confidence interval, IDI: integrated discrimination improvement, NRI: net reclassification improvement.

Table S9. Subgroup analysis for cumCTI-depression and CVD risk.

Characteristics	Number	A	B		C		D		*p* for interaction
		ref	HR (95%CI)	*p*	HR (95%CI)	*p*	HR (95%CI)	*p*	
**Age**									0.55
<60	275	ref	0.8 (0.24–2.68)	0.72	1.47 (0.60–3.62)	0.40	1.12 (0.39–3.21)	0.84	
⩾60	5367	ref	1.71 (1.42–2.07)	<0.01	1.33 (1.13–1.57)	<0.01	1.82 (1.51–2.19)	<0.01	
**Gender**									0.89
Male	2574	ref	1.83 (1.39–2.42)	<0.01	1.39 (1.10–1.75)	<0.01	1.84 (1.38–2.47)	<0.01	
Female	3068	ref	1.56 (1.21–2.01	<0.01	1.32 (1.05–1.66)	<0.01	1.76 (1.38–2.23)	<0.01	
**Smoke**									0.98
Never	3253	ref	1.67 (1.30–2.15)	<0.01	1.33 (1.06–1.66)	<0.01	1.77 (1.39–2.25)	<0.01	
Ever or current	2389	ref	1.67 (1.26–2.21)	<0.01	1.37 (1.08–1.74)	<0.01	1.84 (1.39–2.45)	<0.01	
**Drink**									0.15
Never	3,667	ref	1.41 (1.12–1.78)	<0.01	1.24 (1.01–1.51)	<0.01	1.68 (1.35–2.08)	<0.01	
Ever or current	1,975	ref	2.36 (1.72–3.24)	<0.01	1.57 (1.18–2.08)	<0.01	2.08 (1.48–2.92)	<0.01	
**Hypertension**									0.37
No	4,200	ref	1.71 (1.36–2.13)	<0.01	1.39 (1.13–1.7)	<0.01	1.96 (1.55–2.47)	<0.01	
Yes	1,442	ref	1.54 (1.1–2.16)	<0.01	1.19 (0.91–1.55)	<0.01	1.52 (1.13–2.05)	<0.01	
**Dyslipidemia**									0.47
No	5,117	ref	1.63 (1.34–1.98)	<0.01	1.32 (1.11–1.57)	<0.01	1.89 (1.55–2.3)	<0.01	
Yes	525	ref	1.96 (1.06–3.6)	0.03	1.4 (0.86–2.29)	0.18	1.52 (0.89–2.6)	0.12	

cumCTI: cumulative C-reactive protein–triglyceride glucose index, CVD: cardiovascular diseases, HR: hazard ratio, CI: confidence interval. A: low cumCTI without depression, B: low cumCTI with depression, C: high cumCTI without depression, D: high cumCTI with depression.

Table S10. Sensitivity analysis for cumCTI-depression and CVD risk with 1%-99% winsorization.

Term	HR	95%CI	*p*-value
Low cumCTI with depression	1.74	1.44 -2.10	<0.01
High cumCTI without depression	1.35	1.15 - 1.59	<0.01
High cumCTI with depression	1.83	1.53 - 2.20	<0.01
Age	1.03	1.02 - 1.03	<0.01
Gender (female)	1.27	1.04 - 1.55	0.02
Marital status (other)	0.96	0.82 - 1.13	0.63
Smoke (yes)	1.26	1.05 - 1.53	0.02
Drink (yes)	0.93	0.81 - 1.08	0.34
Night sleeping time	0.99	0.96 - 1.02	0.43
Nap time	1.00	1.00 - 1.00	0.27
Hypertension (yes)	1.67	1.47 - 1.90	<0.01
Dyslipidemia (yes)	1.66	1.40 - 1.96	<0.01
Dysglycemia (yes)	1.39	1.13 - 1.70	<0.01
Kidney disease (yes)	1.16	0.89 - 1.50	0.28
Psychiatric problems (yes)	1.88	1.11 - 3.20	0.02

cumCTI: cumulative C-reactive protein–triglyceride glucose index, CVD: cardiovascular diseases, , HR: hazard ratio, CI: confidence interval.

Table S11. Sensitivity analysis for cumCTI-depression and CVD risk with multiple imputation.

**Term**	**HR**	**95%CI**	***p*-value**
High cumCTI with depression	1.81	1.50 - 2.18	<0.01
High cumCTI without depression	1.34	1.13 - 1.58	<0.01
Low cumCTI with depression	1.76	1.45 - 2.13	<0.01
Age	1.03	1.02 - 1.03	<0.01
Gender (female)	1.30	1.06 - 1.58	0.01
Marital status (other)	0.94	0.80 - 1.11	0.47
Smoke (yes)	1.28	1.06 - 1.55	0.01
Drink (yes)	0.94	0.81 - 1.08	0.39
Night sleeping time	0.98	0.95 - 1.02	0.33
Nap time	1.00	1.00 - 1.00	0.22
Hypertension (yes)	1.73	1.51 - 1.97	<0.01
Dyslipidemia (yes)	1.66	1.39 - 1.98	<0.01
Dysglycemia (yes)	1.29	1.04 - 1.59	0.02
Kidney disease (yes)	1.20	0.93 - 1.56	0.17
Psychiatric problems (yes)	1.80	1.05 - 3.09	0.03

cumCTI: cumulative C-reactive protein–triglyceride glucose index, CVD: cardiovascular diseases, HR: hazard ratio, CI: confidence interval.

Table S12. Cox proportional hazards models of the associations between joint exposure categories and CVD risk using depressive symptom trajectories

	**Model 1**		**Model 2**		**Model 3**		**Model 4**	
**Group**	HR (95%CI)	*p*	HR (95%CI)	*p*	HR (95%CI)	*p*	HR (95%CI)	*p*
Low cumCTI without depression	ref	ref	ref	ref	ref	ref	ref	ref
Low cumCTI with remitted depression	0.93 (0.69–1.25)	0.626	0.90 (0.67–1.21)	0.466	0.89 (0.66–1.19)	0.431	0.89 (0.66–1.20)	0.435
Low cumCTI with new-onset depression	1.57 (1.22–2.03)	<0.001	1.65 (1.27–2.13)	<0.001	1.61 (1.24–2.08)	<0.001	1.60 (1.23–2.07)	<0.001
Low cumCTI with persistent depression	1.83 (1.46–2.29)	<0.001	1.84 (1.47–2.31)	<0.001	1.81 (1.44–2.28)	<0.001	1.79 (1.42–2.25)	<0.001
High cumCTI without depression	1.42 (1.18–1.71)	<0.001	1.43 (1.19–1.72)	<0.001	1.41 (1.18–1.70)	<0.001	1.22 (1.02–1.48)	<0.001
High cumCTI with remitted depression	2.01 (1.59–2.55)	<0.001	1.95 (1.54–2.47)	<0.001	1.91 (1.51–2.42)	<0.001	1.64 (1.29–2.09)	<0.001
High cumCTI with new-onset depression	1.99 (1.56–2.52)	<0.001	1.98 (1.56–2.52)	<0.001	1.93 (1.52–2.46)	<0.001	1.66 (1.30–2.12)	<0.001
High cumCTI with persistent depression	2.56 (2.07–3.17)	<0.001	2.47 (1.99–3.07)	<0.001	2.39 (1.92–2.98)	<0.001	1.93 (1.54–2.41)	<0.001

cumCTI: cumulative C-reactive protein–triglyceride glucose index, CVD: cardiovascular diseases, HR: hazard ratio, CI: confidence interval.

Model 1: unadjusted

Model 2: adjusted for age, gender, and marital status

Model 3: further adjusted for smoke, drink, night sleeping time, and nap time

Model 4: further adjusted for hypertension, dyslipidemia, dysglycemia, kidney disease, and psychiatric problems

Table S13. Cox proportional hazards models of the association between cumCTI-depression categories and CVD risk using midpoint-imputed event times

	Model 1		Model 2		Model 3		Model 4	
**Group**	HR (95%CI)	*p*	HR (95%CI)	*p*	HR (95%CI)	*p*	HR (95%CI)	*p*
Low cumCTI without depression	ref	ref	ref	ref	ref	ref	ref	ref
Low cumCTI with depression	1.74 (1.45–2.09)	<0.001	1.79 (1.49–2.15)	<0.001	1.75 (1.45–2.11)	<0.001	1.74 (1.44–2.09)	<0.001
High cumCTI without depression	1.57 (1.34–1.84)	<0.001	1.58 (1.35–1.85)	<0.001	1.56 (1.33–1.83)	<0.001	1.35 (1.15–1.59)	0.003
High cumCTI with depression	2.31 (1.94–2.75)	<0.001	2.28 (1.91–2.72)	<0.001	2.21 (1.85–2.64)	<0.001	1.83 (1.53–2.20)	<0.001

cumCTI: cumulative C-reactive protein–triglyceride glucose index, CVD: cardiovascular diseases, HR: hazard ratio, CI: confidence interval.

Model 1: unadjusted

Model 2: adjusted for age, gender, marital status,

Model 3: further adjusted for smoke, drink, night sleeping time, and nap time

Model 4: further adjusted for hypertension, dyslipidemia, dysglycemia, kidney disease, and psychiatric problems

Table S14. Cox proportional hazards models of the association between cumCTI-depression categories and heart disease risk

	Model 1		Model 2		Model 3		Model 4	
**Group**	HR (95%CI)	*p*	HR (95%CI)	*p*	HR (95%CI)	*p*	HR (95%CI)	*p*
Low cumCTI without depression	ref	ref	ref	ref	ref	ref	ref	ref
Low cumCTI with depression	1.78 (1.44–2.21)	<0.001	1.79 (1.44–2.21)	<0.001	1.70 (1.37–2.12)	<0.001	1.52 (1.22–1.89)	<0.001
High cumCTI without depression	1.51 (1.25–1.82)	<0.001	1.50 (1.24–1.81)	<0.001	1.49 (1.23–1.80)	<0.001	1.30 (1.07–1.57)	0.008
High cumCTI with depression	1.99 (1.61–2.45)	<0.001	1.89 (1.53–2.34)	<0.001	1.79 (1.45–2.23)	<0.001	1.67 (1.35–2.08)	<0.001

cumCTI: cumulative C-reactive protein–triglyceride glucose index, CVD: cardiovascular diseases, HR: hazard ratio, CI: confidence interval.

Model 1: unadjusted

Model 2: adjusted for age, gender, marital status

Model 3: further adjusted for smoke, drink, night sleeping time, and nap time

Model 4: further adjusted for hypertension, dyslipidemia, dysglycemia, kidney disease, and psychiatric problems

Table S15. Cox proportional hazards models of the association between cumCTI-depression categories and stroke risk

	Model 1		Model 2		Model 3		Model 4	
**Group**	HR (95%CI)	*p*	HR (95%CI)	*p*	HR (95%CI)	*p*	HR (95%CI)	*p*
Low cumCTI without depression	ref	ref	ref	ref	ref	ref	ref	ref
Low cumCTI with depression	1.74 (1.28–2.37)	<0.001	1.90 (1.40–2.59)	<0.001	1.92 (1.41–2.62)	<0.001	1.91 (1.40–2.60)	<0.001
High cumCTI without depression	1.74 (1.34–2.26)	<0.001	1.80 (1.38–2.34)	<0.001	1.77 (1.36–2.30)	<0.001	1.49 (1.14–1.95)	0.003
High cumCTI with depression	3.04 (2.31–4.00)	<0.001	3.24 (2.45–4.27)	<0.001	3.22 (2.43–4.26)	<0.001	2.60 (1.95–3.46)	<0.001

cumCTI: cumulative C-reactive protein–triglyceride glucose index, CVD: cardiovascular diseases, HR: hazard ratio, CI: confidence interval.

Model 1: unadjusted

Model 2: adjusted for age, gender, marital status

Model 3: further adjusted for smoke, drink, night sleeping time, and nap time

Model 4: further adjusted for hypertension, dyslipidemia, dysglycemia, kidney disease, and psychiatric problems

Table S16. Survey-weighted Cox proportional hazards models of the association between cumCTI-depression categories and CVD risk

	**Model 1**		**Model 2**		**Model 3**		**Model 4**	
**Group**	HR (95%CI)	*p*	HR (95%CI)	*p*	HR (95%CI)	*p*	HR (95%CI)	*p*
Low cumCTI without depression	ref	ref	ref	ref	ref	ref	ref	ref
Low cumCTI with depression	1.72 (1.39–2.13)	<0.001	1.77 (1.43–2.18)	<0.001	1.74 (1.40–2.16)	<0.001	1.75 (1.41–2.18)	<0.001
High cumCTI without depression	1.51 (1.24–1.83)	<0.001	1.49 (1.23–1.80)	<0.001	1.47 (1.21–1.79)	<0.001	1.29 (1.06–1.56)	0.011
High cumCTI with depression	2.33 (1.92–2.84)	<0.001	2.27 (1.86–2.77)	<0.001	2.22 (1.82–2.71)	<0.001	1.87 (1.52–2.31)	<0.001

cumCTI: cumulative C-reactive protein–triglyceride glucose index, CVD: cardiovascular diseases, HR: hazard ratio, CI: confidence interval.

Model 1: unadjusted

Model 2: adjusted for age, gender, marital status

Model 3: further adjusted for smoke, drink, night sleeping time, and nap time

Model 4: further adjusted for hypertension, dyslipidemia, dysglycemia, kidney disease, and psychiatric problems

Table S17. Cox proportional hazards models of the association between cumCTI-depression categories and CVD risk with additional covariates

	**Model 1**		**Model 2**		**Model 3**		**Model 4**		**Model 5**	
**Group**	HR (95%CI)	*p*	HR (95%CI)	*p*	HR (95%CI)	*p*	HR (95%CI)	*p*	HR (95%CI)	*p*
Low cumCTI without depression	ref	ref	ref	ref	ref	ref	ref	ref	ref	ref
Low cumCTI with depression	1.75 (1.46–2.11)	<0.001	1.81 (1.50–2.18)	<0.001	1.78 (1.47–2.15)	<0.001	1.82 (1.50–2.19)	<0.001	1.77 (1.46–2.14)	<0.001
High cumCTI without depression	1.58 (1.35–1.86)	<0.001	1.59 (1.36–1.87)	<0.001	1.57 (1.34–1.85)	<0.001	1.40 (1.19–1.66)	<0.001	1.29 (1.09–1.52)	0.003
High cumCTI with depression	2.33 (1.96–2.78)	<0.001	2.29 (1.92–2.73)	<0.001	2.23 (1.86–2.66)	<0.001	2.01 (1.67–2.41)	<0.001	1.86 (1.57–2.21)	<0.001

cumCTI: cumulative C-reactive protein–triglyceride glucose index, CVD: cardiovascular diseases, HR: hazard ratio, CI: confidence interval

Model 1: unadjusted

Model 2: adjusted for age, gender, marital status, education group, economic state, and residence

Model 3: further adjusted for physical activity level, smoke, drink, night sleeping time and nap time

Model 4: further adjusted for BMI

Model 5: further adjusted for hypertension, dyslipidemia, dysglycemia, kidney disease, psychiatric problems, antihypertensive, lipid-lowering, and antidiabetic medications
